# Common Variants in *CYP2R1* and *GC* Genes Predict Vitamin D Concentrations in Healthy Danish Children and Adults

**DOI:** 10.1371/journal.pone.0089907

**Published:** 2014-02-27

**Authors:** Janna Nissen, Lone Banke Rasmussen, Gitte Ravn-Haren, Elisabeth Wreford Andersen, Bettina Hansen, Rikke Andersen, Heddie Mejborn, Katja Howarth Madsen, Ulla Vogel

**Affiliations:** 1 Division of Nutrition, National Food Institute, Technical University of Denmark, Søborg, Denmark; 2 Division of Toxicology and Risk Assessment, National Food Institute, Technical University of Denmark, Søborg, Denmark; 3 Department of Applied Mathematics and Computer Science, Technical University of Denmark, Lyngby, Denmark; 4 Department of Biomedicine, Aarhus University, Aarhus, Denmark; 5 National Research Centre for the Working Environment, Copenhagen, Denmark; University of Illinois at Chicago, United States of America

## Abstract

Environmental factors such as diet, intake of vitamin D supplements and exposure to sunlight are known to influence serum vitamin D concentrations. Genetic epidemiology of vitamin D is in its infancy and a better understanding on how genetic variation influences vitamin D concentration is needed. We aimed to analyse previously reported vitamin D-related polymorphisms in relation to serum 25(OH)D concentrations in 201 healthy Danish families with dependent children in late summer in Denmark. Serum 25(OH)D concentrations and a total of 25 SNPs in *GC*, *VDR*, *CYP2R1*, *CYP24A1*, *CYP27B1*, *C10or88* and *DHCR7/NADSYN1* genes were analysed in 758 participants. Genotype distributions were in Hardy–Weinberg equilibrium for the adult population for all the studied polymorphisms. Four SNPs in *CYP2R1* (rs1562902, rs7116978, rs10741657 and rs10766197) and six SNPs in *GC* (rs4588, rs842999, rs2282679, rs12512631, rs16846876 and rs17467825) were statistically significantly associated with serum 25(OH)D concentrations in children, adults and all combined. Several of the SNPs were in strong linkage disequilibrium, and the associations were driven by *CYP2R1*-rs10741657 and rs10766197, and by *GC*-rs4588 and rs842999. Genetic risk score analysis showed that carriers with no risk alleles of *CYP2R1*-rs10741657 and rs10766197, and/or *GC* rs4588 and rs842999 had significantly higher serum 25(OH)D concentrations compared to carriers of all risk alleles. To conclude, our results provide supporting evidence that common polymorphisms in *GC* and *CYP2R1* are associated with serum 25(OH)D concentrations in the Caucasian population and that certain haplotypes may predispose to lower 25(OH)D concentrations in late summer in Denmark.

## Introduction

Vitamin D deficiency is a widespread problem in developed countries [1]. Severe vitamin D deficiency causes osteomalacia, or childhood rickets, osteoporosis and fractures because of reduced calcium absorption [2]. Low vitamin D concentrations may also be related to various non-skeletal health outcomes, including cardiovascular diseases [3], obesity [4], diabetes [5], asthma [6], multiple sclerosis [7], occurrence of a large range of cancer diseases [8] and overall mortality [9], [10].

In humans, vitamin D is produced mainly in the skin during exposure to solar ultraviolet blue (UVB) radiation (270–300 nm) [11]. UVB radiation converts 7-dehydrocholesterol (7-DHC) in the skin to pre-vitamin D_3_, which immediately undergoes a thermal isomerization to vitamin D_3_. Dietary sources provide two forms of vitamin D: Vitamin D_2_ (ergocalciferol) derived from invertebrates (plants and fungi) and vitamin D_3_ (cholecalciferol) derived from animal sources. Ingested vitamins D_2_ and D_3_ are absorbed in the small intestine and transported with chylomicrons and lipoproteins to the liver, whereas dermally synthesized vitamin D_3_ diffuses via the blood to the liver tightly bound to group-specific complement (GC) [12].

Dietary or dermally synthesized vitamin D (hereafter “D” refers to D_2_ and D_3_) undergoes a series of enzymatic conversions in the liver and kidneys to become biologically active. The hepatic enzyme 25-hydroxylase (CYP2R1) converts vitamin D to 25-hydroxyvitamin D (25(OH)D). This is the major circulating form of vitamin D in the blood. To become biologically active, 25(OH)D is converted to 1,25-dihydroxyvitamin D (1,25(OH)_2_D). This occurs mainly in the kidneys, but also in other tissues expressing the enzyme 25(OH)D-1α-hydroxylase (CYP27B1). The biological effect of vitamin D is mediated when 1,25(OH)_2_D binds to the vitamin D receptor (VDR). To prevent excessive vitamin D signalling in the target organs, 1,25(OH)_2_D limits its own activity by inducing 24-hydroxylase (CYP24A1) converting 1,25(OH)_2_D to the biologically inactive water-soluble calcitroic acid which is excreted in the bile [1], [12], [13].

The best biomarker of vitamin D concentration is the serum 25(OH)D concentration. Approximately 25% of the inter-individual variability in plasma 25(OH)D concentrations can be explained by external factors such as diet, regular use of vitamin D supplements and exposure to sunlight (dependent on season and latitude) [14], [15]. Genetic factors may contribute to vitamin D concentrations. Results from twin and family-based studies indicate that blood vitamin D concentrations to some extent are under genetic control. The results have been inconsistent with a wide variability in heritability estimates ranging from 23 to 80% [15]–[21]. Furthermore, ethnic differences in vitamin D concentrations have also been described [22].

Genetic epidemiology of vitamin D is in its infancy and a better understanding of how genetic variation influences vitamin D concentrations is needed. A growing number of studies have uncovered polymorphisms associated with vitamin D concentrations. By candidate gene analysis, five genes have been found, including *GC, CYP24A1, CYP2R1, CYP27B1* and *VDR* [23]. Recently, two genome-wide association studies (GWAS) of vitamin D [24], [25] confirmed the associations of common variants in *GC* and *CYP2R1* genes. Furthermore, nicotinamide adenine dinucleotide synthetase-1/7-dehydrocholesterol reductase (NADSYN1/DHCR7), and the region harbouring the open-reading frame 88 (C10orf88) on chromosome 10q26.13 were also found to be associated with vitamin D concentrations in blood.

In Denmark, low vitamin D status is common during the winter due to inadequate dietary intakes and lack of solar radiation from September to April [26]. We assessed vitamin D status in late summer (September to October), where the Danes vitamin D concentration peaks but are not saturated [27], in families with a broad span in age in both children and adults. In children, the role of genetic variation in determining serum 25(OH)D concentrations is an understudied area.

In this study, we analysed previously reported vitamin D-related polymorphisms in relation to serum 25(OH)D concentrations in 201 healthy Danish families with dependent children to confirm previous findings and thus help identifying individuals that may have increased risk of developing vitamin D insufficiency.

## Subjects and Methods

### Study population

The present cross-sectional study used baseline data from the VitmaD intervention study described in detail elsewhere [28]. Briefly, 201 Danish families with dependent children (n = 782) were enrolled. The participants were 4- to 60-years old. Baseline blood samples were collected in September and October 2010 and were obtained from 770 participants. The study was conducted according to the guidelines in the Declaration of Helsinki and the protocol was approved by the Research Ethics Committee of the Capital Region of Denmark (H-4-2010-020) and registered at http://clinicaltrials.gov (NCT01184716). All adult participants and guardians on the behalf of the children participants gave written consent to participate.

### DNA extraction and genotyping

DNA was extracted from peripheral blood leukocytes as described by Miller *et al*. [29] and stored in TE-buffer at -80°C. The DNA was diluted to 10 ng/µl using a Nanodrop® ND-1000 Spectrophotometer (Thermo Fisher Scientific Inc., Wilmington). Single nucleotide polymorphisms (SNPs) were genotyped using the Sequenom MassARRAY iPLEX Gold platform (Sequenom, San Diego, California) at the Department of Biomedicine, Aarhus University, Denmark. Genotyping was successful for 762 participants (99.0%). To confirm the accuracy of genotyping duplicate samples (10%) yielded 100% reproducibility.

All SNPs were located in or near genes involved in vitamin D synthesis, activation or degradation. The following SNPs were selected on the basis of evidence of significant association in previous studies: ***CYP2R1*** (rs1562902; rs7116978; rs10741657; rs10766197) ***CYP24A1*** (rs229624; rs2426496; rs4809960; rs6013897; rs17219315) ***CYP27B1*** (rs10877012) ***C10orf88*** (rs6599638) ***DHCR7/NADSYN1*** (rs1790349; rs12785878) ***GC*** (rs4588; rs222020; rs842999-triallelic; rs2882679; rs2298849; rs12512631; rs16846876; rs17467825) ***VDR*** (rs731236 (TaqI), rs757343 (TruI); rs7139166; rs10783219).

Deviation from Hardy–Weinberg equilibrium (HWE) was tested for the adult population using Chi-square test with Bonferroni’s correction (P-value 0.05/25 SNPs = 0.002). No significant deviation from HWE was observed. Linkage disequilibrium (LD) between polymorphisms was evaluated using Pearsons’ r, SNAP version 2.2 (http://www.broadinstitute.org/mpg/snap/ldsearchpw.php) and Haploview software version 4.2 for the adult population.

### Measurement of serum 25(OH)D concentrations

Measurements of serum 25(OH)D concentrations are described in detail elsewhere [28]. Briefly, blood samples were obtained without prior fasting and serum was stored in aliquots at –80°C until analysis. Measurements of serum 25(OH)D concentrations relied on the determination of both 25(OH)D_2_ and 25(OH)D_3_ and were conducted by isotope dilution liquid chromatography tandem mass spectrometry (LC-MS/MS) at Clinical Biochemical Department, Holbæk Hospital, Denmark. As primary calibrator the standard reference material, vitamin D in humans (SRM 972) from the National Institute of Standards and Technology was used. The analytic quality of 25(OH)D assay was assured by Vitamin D External Quality Assessment Scheme certification and the mean bias was –3.2%. The Inter-assay CVs for 25(OH)D_2_ were 7.6% and 4.6% at 43 and 150 nmol/L, respectively, and for 25(OH)D_3_ 2.2% and 2.8% at 30 and 180 nmol/L, respectively [28]. Of the 762 participants that were successfully genotyped, baseline serum 25(OH)D concentrations were measured for 758 participants.

### Statistical analysis

Statistical analyses were performed using SAS Enterprise Guide 4.3 (SAS Institute, Inc., Cary. USA). Serum 25(OH)D concentrations were log transformed to approximate a normal distribution and all means are presented as geometric means. A nominal P-value of 0.05 was considered statistically significant. Linear mixed models with family as a random factor were applied to account for the possible dependence between the participants. Furthermore, in the linear mixed models the following categorical variables were included: age (4–11, 12–17, 18–40, 41–60 years), sex (male, female), BMI (underweight, normal weight, overweight, obese) according to standards for children [30] and the WHO International standards for adults [31], ski or sun holidays (yes, no), solarium use at least once a week (yes, no), dietary vitamin D (quartiles: <1.7, 1.7–2.4, 2.5–3.3 and >3.3 µg/d), multivitamin and vitamin D supplement users (yes, no). The data were obtained from a self-administered web-based questionnaire and a semi-quantitative food frequency questionnaire based on the last six months. Pearson’s r were calculated on the adult population and were used to assess the degree of linkage between linked SNPs. Haplotypes were inferred manually among the adults, only since the children were not population-based. The inferred haplotype combinations described 100% and 97% of the observed genotypes among the adults for *CYP2R1* and *GC* genes, respectively. Among the children the inferred haplotype combinations described 100% and 96% of the observed genotypes for *CYP2R1* and *GC* genes, respectively. Each derived haplotype was assigned a number. Homozygote haplotype combinations were numbered with two identical numbers e.g. 11. The combinations of heterozygote haplotypes were given by the combination of the number of each haplotype e.g. 1+ 2 = 12.

Genetic risk scores were calculated as the sum of risk alleles and included as risk factors in linear mixed models adjusted for family and confounding variables. The correlation coefficient for rs10741657, rs10766197, rs4588 and rs842999 were very similar and therefore it was not necessary to weight the score by effect size. All the analyses were performed separately for children, adults and for all combined.

## Results

Genotyping and serum 25(OH)D concentrations were available for 758 participants. Table 1 summarizes the basic characteristics of the study population, previously described in detail elsewhere [28]. The median age among children was 10 years (range: 4 to17) among adults 41 years (range: 18 to 60) and for all combined 30 years.

**Table 1:** Basic characteristics of the study population and determinants of serum 25(OH)D concentrations

Characteristics	Children	Adults	All
Number	348	414	762
Female/Male (n/n)	181/167	209/205	390/372
Age, median (range)	10 (4–17)	41 (18–60)	30 (4–60)
BMI (kg/m^2^)*	17.44±2.89	25.47±4.30	21.79±5.45
Serum 25(OH)D (nmol/L)*	74.38±17.31	74.87±21.70	74.65±19.82
Dietary Vitamin D (μg/d)*	2.69±1.35	2.96±2.04	2.84±1.77
Multivitamin or vitamin D supplement users (yes/no)	141/203	113/297	254/500
Solarium use (yes/no)	2/342	10/401	12/743
Ski or sun holidays (yes/no)	195/149	220/191	415/340

*Mean ± SD.

Associations between genotypes and serum 25(OH)D concentrations are shown for children, adults and all combined in Table 2. After adjustment for family and confounding factors, all four analysed SNPs in *CYP2R1* were statistically significantly associated with serum 25(OH)D concentrations in all three groups. Furthermore, for all three groups none of the analysed SNPs in *CYP24A1, CYP27B1*, *C10orf88* and *DHCR7/NADSYN1* were statistically significantly associated with serum 25(OH)D concentration. For all three groups all analysed SNPs in *GC*, except rs2298849 (in all three groups) and rs222020 (in adults and all), were statistically significantly associated with serum 25(OH)D concentration. The *VDR* rs731236 was only statistically significantly associated with 25(OH)D concentration in all combined and rs757343 was statistically significant in children and all combined. Only SNPs that were statistically significantly associated with 25(OH)D concentrations in children, adults and all combined were included in further analyses.

**Table 2:** Basic characteristics of the individual SNP and the association with serum 25(OH)D concentrations in children, adults and all combined.

					Children (n = 344)				Adults (n = 414)				All (n = 758)			
SNP	MMAF	HWE	M/m	Gt	N	25(OH)D,	p^1^	p_adj_^2^	N	25(OH)D,	p^1^	p_adj_^2^	N	25(OH)D,	p^1^	p_adj_^2^
						nmol/L				nmol/L				nmol/L		
						(95% CI)				(95% CI)				(95% CI)		
***CYP2R1***																
**rs7116978**	38.8	0.25	C/T	CC	124	67.6 (65.0–70.2)	**<0.0001**	**<0.0001**	156	67.5 (64.2–71.0)	**0.0218**	**0.0093**	280	67.5 (65.3–69.8)	**<0.0001**	**<0.0001**
				CT	158	73.9 (71.4–76.6)			180	72.8 (69.5–76.3)			338	73.3 (71.2–75.6)		
				TT	54	79.1 (74.5–83.9)			66	77.5 (71.8–83.8)			120	78.2 (74.4–82.3)		
**rs10741657**	40.8	0.31	G/A	GG	118	67.9 (65.2–70.7)	**<0.0001**	**<0.0001**	150	66.6 (63.3–70.1)	**0.0039**	**0.0067**	268	67.2 (65.0–69.5)	**<0.0001**	**<0.0001**
				GA	175	73.9 (71.5–76.4)			190	74.0 (70.7–77.4)			365	73.9 (71.8–76.1)		
				AA	51	78.8 (74.1–83.7)			74	75.2 (69.9–80.9)			125	76.6 (73.0–80.5)		
**rs1562902**	45.2	0.37	T/C	TT	103	68.9 (65.9–71.9)	**0.0233**	**0.0086**	129	67.5 (63.9–71.4)	**0.0574**	**0.0353**	232	68.1 (65.7–70.6)	**0.0022**	**0.0005**
				TC	172	73.7 (71.2–76.2)			196	73.3 (70.0–76.6)			368	73.5 (71.4–75.6)		
				CC	69	75.0 (71.0–79.1) 79.1			89	73.4 (68.6–78.5)			158	74.1 (70.9–77.4)		
**rs10766197**	46.9	0.15	G/A	GG	97	76.0 (72.7–79.5)	**0.0048**	**0.0006**	124	73.0 (69.0–77.3)	0.0557	**0.0081**	221	74.3 (71.6–77.1)	**0.0013**	**<0.0001**
				AG	168	72.7 (70.2–75.2)			191	73.2 (69.9–76.6)			359	72.9 (70.8–75.1)		
				AA	79	67.9 (64.6–71.4)			98	66.2 (62.1–70.5)			177	66.9 (64.2–69.8)		
***CYP24A1***																
**rs6013897**	20.3	0.77	T/A	TT	219	73.5 (71.3–75.8)	0.2887	0.5044	264	71.8 (69.1–74.7)	0.9033	0.7058	483	72.6 (70.8–74.4)	0.4702	0.5228
				AT	114	70.7 (67.8–73.8)			132	70.9 (67.1–74.9)			246	70.8 (68.4–73.4)		
				AA	11	69.5 (60.7–79.5)			18	70.0 (60.3–81.3)			29	69.8 (63.0–77.4)		
**rs4809960**	22.7	0.35	T/C	TT	198	72.0 (69.7–74.3)	0.8163	0.5674	244	72.2 (69.3–75.1)	0.3402	0.2786	442	72.1 (70.2–74.0)	0.4658	0.0663
				TC	121	72.9 (70.0–76.0)			152	69.7 (66.2–73.3)			273	71.1 (68.7–73.5)		
				CC	25	73.8 (67.5–80.7)			18	77.2 (66.5–89.6)			43	75.2 (69.1–81.9)		
**rs2296241**	49.0	0.37	G/A	GG	90	68.9 (65.8–72.2)	**0.0301**	0.1111	103	70.3 (66.0–74.8)	0.6048	0.6078	193	69.6 (66.9–72.5)	0.0801	0.0501
				AG	164	72.9 (70.4–75.4)			216	71.1 (68.1–74.3)			380	71.9 (69.9–74.0)		
				AA	90	75.4 (71.9–79.0)			95	73.5 (68.8–78.4)			185	74.4 (71.4–77.5)		
**rs17219315**	3.1	0.75	A/G	AA	342	72.3 (70.6–74.1)	0.0895	0.1836	401	71.4 (69.1–73.7)	0.6621	0.3828	743	71.8 (70.3–73.3)	0.3674	0.2381
				AG	2	95.4 (69.5–130.9)			13	74.3 (62.3–88.6)			15	76.8 (66.5–88.7)		
**rs2426496**	27.7	0.51	G/T	GG	176	71.3 (68.9–73.8)	0.3094	0.2500	214	70.5 (67.5–73.6)	0.6377	0.7896	390	70.8 (68.9–72.9)	0.2573	0.2500
				GT	135	73.2 (70.4–76.0)			171	72.3 (68.9–75.9)			306	72.7 (70.4–75.0)		
				TT	33	75.8 (70.1–81.9)			29	73.9 (65.7–83.1)			62	74.9 (69.8–80.4)		
***CYP27B1***																
**rs10877012**	33.5	0.02	G/T	GG	156	72.8 (70.2–75.4)	0.1846	0.5758	193	71.0 (67.9–74.4)	0.7822	0.9451	349	71.8 (69.7–74.0)	0.3792	0.9918
				GT	142	73.4 (70.7–76.2)			163	72.4 (68.9–76.0)			305	72.9 (70.6–75.2)		
				TT	46	68.4 (64.1–73.1)			57	69.9 (64.3–76.0)			103	69.2 (65.5–73.2)		
***C10orf88***																
**rs6599638**	47.8	0.20	G/A	GG	98	72.5 (69.3–75.8)	0.3569	0.3197	106	72.0 (67.7–76.6)	0.8394	0.8797	204	73.3 (69.5–75.1)	0.8349	0.8821
				GA	171	73.5 (71.0–76.0)			219	70.8 (67.8–73.9)			390	71.9 (69.9–74.0)		
				AA	75	70.2 (66.6–73.9)			88	72.2 (67.4–77.2)			163	71.2 (68.2–74.4)		
***DHCR7/NADSYN1***																
**rs1790349**	15.1	0.55	A/G	AA	232	71.6 (69.6–73.7)	**0.0174**	0.0923	300	70.9 (68.4–73.6)	0.2381	0.3478	532	71.2 (69.5–73.0)	0.3767	0.8787
				GA	105	73.2 (70.1–76.4)			103	73.9 (69.5–78.7)			208	73.6 (70.8–76.5)		
				GG	7	91.5 (77.4–108.3)			11	63.2 (52.2–76.5)			18	73.0 (64.0–83.2)		
**rs12785878**	27.5	0.84	T/G	TT	171	72.8 (70.4–75.4)	0.9087	0.7649	218	73.0 (69.9–76.2)	0.4356	0.2169	389	72.9 (70.9–75.0)	0.4273	0.0998
				GT	147	72.1 (69.5–74.9)			163	69.6 (66.2–73.1)			310	70.8 (68.6–73.1)		
				GG	26	71.7 (65.7–78.4)			32	69.9 (62.5–78.2)			58	70.7 (65.7–76.1)		
***GC***																
**rs16846876**	33.2	0.88	A/T	AA	158	76.5 (73.9–79.2)	**<0.0001**	**0.0004**	184	74.1 (70.7–77.6)	**0.0161**	**0.0024**	342	75.2 (73.0–77.4)	**<0.0001**	**<0.0001**
				AT	153	70.3 (67.8–72.8)			185	70.9 (67.7–74.3)			338	70.6 (68.5–72.8)		
				TT	33	64.5 (59.8–69.6)			45	63.6 (57.9–69.8)			78	64.0 (60.1–68.1)		
**rs12512631**	36.2	0.62	T/C	TT	137	68.6 (66.1–71.2)	**0.0007**	**0.0012**	166	66.8 (63.6–70.1)	**0.0022**	**0.0004**	303	67.6 (65.5–69.8)	**<0.0001**	**<0.0001**
				TC	157	74.4 (71.8–77.1)			196	74.6 (71.3–78.0)			353	74.5 (72.4–76.7)		
				CC	50	77.5 (72.8–82.5)			52	75.3 (69.0–82.1)			102	76.4 (72.3–80.6)		
**rs17467825**	27.6	0.53	A/G	AA	181	76.3 (73.9–78.8)	**<0.0001**	**<0.0001**	219	73.8 (70.7–77.0)	0.0519	**0.0015**	400	74.9 (72.9–77.0)	**<0.0001**	**<0.0001**
				GA	142	70.1 (67.6–72.7)			160	70.0 (66.6–73.6)			302	70.1 (67.9–72.3)		
				GG	21	57.7 (52.5–63.3)			34	63.6 (57.1–70.8)			55	61.2 (56.9–65.9)		
**rs2282679**	27.4	0.41	A/C	AA	181	76.3 (73.9–78.8)	**<0.0001**	**<0.0001**	219	73.8 (70.7–77.0)	0.0672	**0.0020**	400	74.9 (72.9–77.0)	**<0.0001**	**<0.0001**
				CA	138	70.0 (76.4–72.6)			156	70.1 (66.6–73.7)			294	70.0 (67.8–72.3)		
				CC	21	57.7 (52.5–63.3)			34	63.6 (57.1–70.8)			55	61.2 (56.9–65.9)		
**rs842999**	4.5	0.65	G/C/A	GG	105	76.7 (73.5–80.0)	**<0.0001**	**<0.0001**	112	74.2 (70.0–78.7)	**0.0114**	**0.0046**	217	75.4 (72.7–78.3)	**<0.0001**	**<0.0001**
				GC	153	72.6 (70.1–75.2)			188	73.7 (70.4–77.1)			341	73.2 (71.1–75.4)		
				CC	57	63.7 (60.2–67.5)			75	66.6 (61.9–71.5)			132	65.3 (62.3–68.5)		
				GA	19	74.3 (67.3–82.1)			23	64.9 (57.0–73.9)			42	69.0 (63.4–75.1)		
				CA	7	76.3 (64.6–89.6)			12	55.8 (46.6–66.9)			19	62.6 (55.2–71.0)		
				AA	0	-			1	75.5 (40.5–140.9)			1	75.5 (43.6–130.8)		
**rs4588**	27.7	0.57	C/A	CC	181	76.3 (73.9–78.8)	**<0.0001**	**<0.0001**	219	74.1 (71.0–77.3)	**0.0167**	**0.0008**	400	75.1 (73.1–77.2)	**<0.0001**	**<0.0001**
				CA	142	70.1 (67.6–72.7)			161	69.7 (66.3–73.2)			303	69.9 (67.7–72.1)		
				AA	21	57.7 (52.5–63.3)			34	63.6 (57.1–70.8)			55	61.2 (56.9–65.9)		
**rs222020**	15.6	0.13	T/C	TT	250	70.5 (68.6–72.5)	**0.0009**	**0.0021**	291	70.5 (67.9–73.1)	**0.1954**	**0.5338**	541	70.5 (68.8–72.2)	**0.0103**	0.0739
				TC	88	78.4 (74.8–82.1)			117	73.2 (69.1–77.6)			205	75.4 (72.5–78.4)		
				CC	6	69.7 (58.3–83.5)			6	86.4 (66.7–111.8)			12	77.6 (66.1–91.1)		
**rs2298849**	20.2	0.57	T/C	TT	229	71.1 (69.1–73.2)	**0.0170**	**0.2204**	262	70.3 (67.6–73.1)	0.4399	0.4591	491	70.7 (69.0–72.5)	**0.0390**	0.2605
				CT	99	75.4 (72.1–78.8)			137	73.4 (69.5–77.5)			236	74.2 (71.6–77.0)		
				CC	15	71.1 (63.4–79.7)			15	73.3 (62.3–86.3)			30	72.2 (65.2–79.9)		
***VDR***																
**rs731236**	40.3	0.18	T/C	TT	113	70.0 (67.1–73.0)	0.1929	0.0753	154	68.9 (65.4–72.5)	0.1499	0.1306	267	69.3 (67.0–71.7)	0.0753	**0.0346**
				TC	181	74.2 (71.8–76.7)			186	72.3 (69.0–75.7)			367	73.2 (71.1–75.4)		
				CC	49	72.0 (67.5–76.7)			74	74.9 (69.6–80.6)			123	73.7 (70.1–77.5)		
**rs757343**	11.5	0.45	G/A	GG	261	73.9 (71.9–76.0)	**0.0134**	**0.0103**	326	72.2 (69.7–74.7)	0.2350	0.0896	587	72.9 (71.3–74.6)	**0.0144**	**0.0025**
				AG	77	68.4 (65.1–72.0)			81	69.6 (64.9–74.7)			158	69.1 (66.1–72.2)		
				AA	6	63.7 (53.1–76.3)			7	59.9 (47.1–76.0)			13	61.6 (52.8–71.9)		
**rs10783219**	36.4	0.10	A/T	AA	147	72.5 (69.8–75.2)	0.9862	0.7067	160	70.1 (66.7–73.7)	0.4908	0.3913	307	71.2 (69.0–73.5)	0.6600	0.2023
				TA	152	72.6 (70.0–75.2)			207	71.8 (68.7–75.0)			359	72.1 (70.0–74.3)		
				TT	45	72.1 (67.4–77.1)			47	74.6 (68.0–81.8)			92	73.4 (69.2–77.8)		
**rs7139166**	43.0	0.48	C/G	CC	114	72.4 (69.5–75.5)	0.6063	0.4251	131	73.2 (69.2–77.3)	0.2755	0.4324	245	72.8 (70.3–75.5)	0.8342	0.7845
				CG	167	71.6 (69.2–74.1)			210	71.7 (68.6–74.8)			377	71.6 (69.6–73.7)		
				GG	62	74.9 (70.8–79.3)			73	67.9 (63.0–73.1)			135	71.0 (67.7–74.5)		

Bold numbers represent significant P values.

*SNP* single nucleotide polymorphism (ordered by position), *MAF* minor allele frequency for the adult population in procent, *HWE* P-values for Hardy-Weinberg equilibrium in the adult population, *M/m* major and minor alleles, *Gt* genotype, *Mean*, raw serum 25(OH)D concentrations were log-transformed to approximate a normal distribution an given as geometric mean (nmol/L), *95% CI* 95%-confident interval.

1 Unadjusted P values.

2 Adjusted P values. Linear mixed models with family as a random factor, adjusted for age, sex, BMI, ski and sun holidays, use of solarium, dietary vitamin D intake, use of multivitamin and vitamin D supplementation.

### Haplotype and genetic risk score analysis of *CYP2R1*

In the adult population, rs10741657-rs7116978 (Pearson’s r  = 0.90), and rs1076697-rs1562902 (Pearson’s r  = –0.86, data not shown) were in strong LD. To establish which of the SNPs had the strongest association to serum 25(OH)D concentrations, we assess the association between one SNP and serum 25(OH)D concentrations while adjusting for the other SNPs, family and confounding factors in a linear mixed model. After adjustment, rs10766197 (p = 0.0846) had the strongest association compared to rs1562902 (p = 0.8211), and rs10741657 (p = 0.2545) had the strongest association compared to rs7116978 (p = 0.3087, data not shown). In further analysis only rs1076697 and rs10741657 were included.

The two *CYP2R1* variants rs10741657 and rs7116978 formed four haplotypes, where haplotype 1 and 2 were most frequent (Table 3). The possible combinations of the four homozygote haplotype are shown in table 3. One genotype combination could be assigned to both haplotype combinations 12 or 34, but based on the observed haplotype frequencies, the most likely combination was 12. After adjustment for family and confounding factors, carriers of 2 copies of the AG-haplotype (haplotype combination 33) had the highest mean serum 25(OH)D concentration (73.8 (60.1–90.6), 72.9 (57.3–92.5) and 81.3 (66.4–99.6) nmol/L) in children, adults and all combined, respectively. In a linear mixed model, only the homozygous haplotype combinations were included and haplotype combination 44 was excluded because only two participants carried this haplotype combination. The homozygous haplotype combinations were significantly associated with serum 25(OH)D concentrations (p = 0.0059, 0.0450 and 0.0007) in children, adults and all combined, respectively.

**Table 3:** Distribution of *CYP2R1* haplotype combinations and serum 25(OH)D concentrations in children, adults and all combined.

				Children (n = 348)				Adults (n = 413)				All (n = 761)			
Haplotype-combination	rs10741657	rs10766197	Alleles^1^		Raw mean	Adj. Mean			Raw mean	Adj. Mean			Raw mean	Adj. Mean	
				N	25(OH)D^2^	25(OH)D^3^	P_adj_	N	25(OH)D^2^	25(OH)D^3^	P_adj_	N	25(OH)D^2^	25(OH)D^3^	P_adj_
					(95% CI)	(95% CI)			(95% CI)	(95% CI)			(95% CI)	(95% CI)	
11	GG	AA	Mm	65	67.3 (63.8–71.1)	64.9 (46.8–89.9)	**0.0059**	81	65.7 (61.3–70.4)	65.2 (52.5–81.1)	**0.0450**	146	66.4 (63.4–69.5)	67.9 (56.0–82.2)	**0.0007**
22	AA	GG	mM	39	80.6 (75.2–86.4)	78.7 (56.9–108.9)		57	74.3 (68.4–80.8)	74.6 (59.2–94.0)		96	76.8 (72.7–81.3)	78.2 (64.5–94.8)	
33	GG	GG	MM	8	68.6 (58.9–80.0)	63.7 (43.7–92.8)		13	70.5 (59.3–83.8)	66.4 (50.4–87.6)		21	69.8 (61.9–78.6)	68.0 (54.5–85.0)	
44	AA	AA	mm	1	50.9 (33.0–78.6)	-		1	79.2 (42.4–147.9)	-		2	63.5 (43.1–93.5)	-	
12*	GA	AG		112	74.2 (71.2–77.3)			119	75.5 (71.3–80.0)			231	74.8 (72.2–77.6)		
13	GG	AG		47	68.5 (64.3–73.1)			56	67.2 (61.8–73.0)			103	67.8 (64.2–71.6)		
23	GA	GG		50	73.8 (69.4–78.5)			54	72.3 (66.4–78.7)			104	73.0 (69.2–77.0)		
14	GA	AA		15	72.1 (64.2–81.0)			16	68.0 (58.2–79.5)			31	69.9 (63.3–77.3)		
24	AA	AG		11	75.5 (66.2–86.1)			16	78.2 (66.9–91.4)			27	77.1 (69.4–85.6)		

Bold numbers represent significant P values.

Haplotype combinations were manually inferred and numbered. Homozygote haplotype combinations were numbered 11, 22, 33 and 44. The combinations of the heterozygote haplotypes (12 to 24) were given by one number of each homozygote haplotype e.g. 11+ 22 = 12.

* Also haplotype combination 34, but the most likely haplotype combination is 12.

1 *M* major allele, *m* minor allele.

2 Raw geometric mean of serum 25(OH)D concentrations (nmol/L) and corresponding 95%-confidence interval.

3 Adjusted geometric mean of 25(OH)D concentrations (nmol/L) ) and corresponding 95%-confidence interval. Linear mixed models with family as a random factor, adjusted for age, sex, BMI, ski and sun holidays, use of solarium, dietary vitamin D intake, use of multivitamin and vitamin D supplements.

adj Adjusted P values. Haplotype combination 44 was excluded in the linear mixed model due to inadequate participants carrying this haplotype combination.

**Figure 1:**
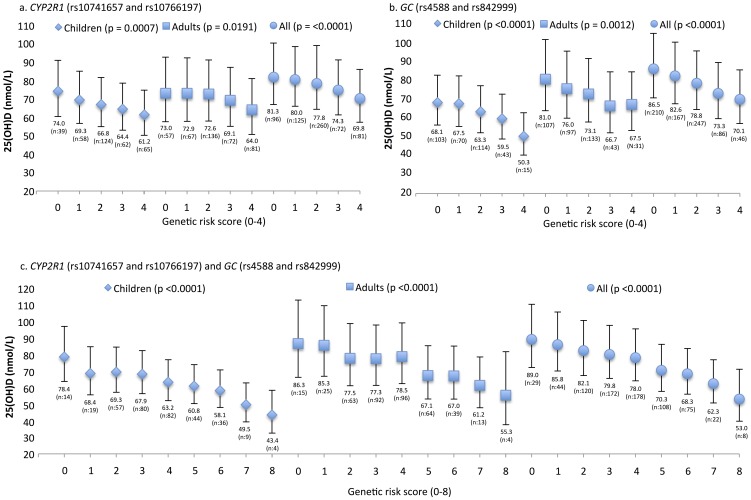
Genetic risk score for *CYP2R1* (rs10741657 and rs10766197) (Figure A), *GC* (r4588 and rs842999) (figure B) and *CYP2R1* (rs10741657 and rs10766197) and *GC* (r4588 and rs842999) (figure C) in children, adults and all combined. X-axis stands for the sum of risk alleles. Y-axis stand for serum 25(OH)D (nmol/L). Errors bars stand for 95%-confidence interval and serum 25(OH)D concentrations are given as geometric means. Linear mixed models with family as a random factor, adjusted for age, sex, BMI, ski and sun holidays, solarium use at least once a week, dietary vitamin D intake, multivitamin and vitamin D supplement users was conducted to compare sum of risk alleles and serum 25(OH)D concentrations. Increasing number of risk alleles give rise to decreasing 25(OH)D concentrations.

We calculated a genetic risk score (range 0–4) as the sum of the number of G-alleles of rs10741657 and A-alleles of rs10766197 (Figure 1, A). After adjustment for family and confounding factors, carriers of no risk alleles had significantly higher serum 25(OH)D concentrations (74.0 (60.3–90.0), 73.0 (57.5–92.6) and 81.3 (66.4–99.5) nmol/L) compared to carriers of all four risk alleles (61.2 (57.5–92.6), 64.0 (50.6–80.9) and 69.8 (57.0–85.4) nmol/L) in children, adults and all combined, respectively. Overall, there was 20.9, 14.1 and 16.5% difference in serum 25(OH)D concentrations between carrying no risk alleles and carrying all four risk alleles in children, adults or all combined, respectively.

### Haplotype and genetic risk score analysis of *GC*

In the adult population, rs4588 was in strong LD with rs2282679 (Pearson’s r = 0.997), rs17467825 (Pearson’s r = 0.997) and rs16846876 (Pearson’s r = 0.805). Furthermore, rs17467825-rs2282679 (Pearson’s r = 1.00), and rs2282679-rs16846876 (Pearson’s r  = 0.8021, data not shown) were also in strong LD. To establish which of the 4 SNPs had the strongest association to serum 25(OH)D concentrations, we assess the association between one SNP and serum 25(OH)D concentrations while adjusting for the other SNPs, family and confounding factors in a linear mixed model. The strongest association was observed for rs4588 (p = 0.0099) compared to rs2282679 (p = 0.0230), rs17467825 (p = 0.0230) and rs16846876 (p = 0.5669, data not shown). Further analyses only included rs4588. None of the other *GC*-variants were in LD.

The three significant *GC*-variants (rs4588, rs842999, and rs12512631) formed five haplotypes, where haplotype 1 and 2 were the most frequent (Table 4). The combinations of the five haplotypes are shown in table 4. The five haplotypes could explain 723 of the 762 (95%) observed genotype combinations in *GC* (data not shown). The association between haplotype combinations and serum 25(OH)D concentrations was statistically significant in children (p = 0.0344), and all combined (p = 0.0018) but not in adults (p = 0.1541).

**Table 4:** Distribution of *GC* haplotype combinations and serum 25(OH)D concentrations in children, adults and all combined.

					Children (n = 215)				Adults (n = 262)				All (n = 488)			
Haplotype-combination	rs12512631	rs842999	rs4588	Alleles^1^		Raw mean	Adj. Mean			Raw mean	Adj. Mean			Raw mean	Adj. Mean	
					N	25(OH)D^2^	25(OH)D^3^	P_adj_	N	25(OH)D^2^	25(OH)D^3^	P_adj_	N	25(OH)D^2^	25(OH)D^3^	P_adj_
						(95% CI)	(95% CI)			(95% CI)	(95% CI)			(95% CI)	(95% CI)	
11	CC	GG	CC	mMM	48	78.0 (73.3–82.9)	86.3 (65.7–106.3)	**0.0344**	49	75.6 (69.4–82.5)	71.8 (48.3–106.8)	0.1541	97	76.8 (72.7–81.1)	88.3 (63.3–123.1)	**0.0018**
22	TT	CC	AA	Mmm	15	56.1 (50.3–62.5)	61.6 (47.1–80.7)		31	65.9 (59.2–73.5)	58.2 (40.8–82.9)		46	62.5 (57.8–67.7)	69.3 (50.3–95.4)	
33	TT	GG	CC	MMM	7	69.2 (59.0–81.2)	74.4 (56.9–97.2)		14	69.7 (59.3–82.0)	64.6 (41.8–99.7)		21	69.6 (61.9–78.2)	79.8 (56.0–113.9)	
44	TT	CC	CC	MmM	8	68.9 (59.4–80.0)	69.7 (53.0–91.8)		9	74.9 (61.2–91.7)	66.3 (40.6–108.3)		17	72.0 (63.2–82.1)	78.6 (54.7–113.1)	
55	TT	AA	CC	MmM	0	–	–		1	75.5 (41.2–138.3)	–		1	75.5 (44.1–129.3)	–	
12	TC	GC	CA		65	71.9 (68.2–75.7)			77	74.2 (69.3–79.5)			142	73.1 (69.9–76.5)		
13	TC	GG	CC		48	76.7 (72.1–81.5)			44	77.5 (70.7–84.9)			92	77.1 (72.8–81.5)		
14	TC	GC	CC		30	78.0 (72.3–84.3)			51	79.3 (72.9–86.3)			81	78.8 (74.3–83.7)		
23	TT	GC	CA		34	70.3 (65.4–75.6)			39	69.6 (63.2–76.7)			73	69.9 (65.7–74.5)		
42	TT	CC	CA		33	66.4 (61.6–71.5)			32	66.6 (59.8–74.1)			65	66.5 (62.2–71.1)		
15	TC	GA	CC		11	70.0 (61.6–79.4)			16	67.0 (57.6–77.9)			27	68.2 (61.5–75.6)		
34	TT	GC	CC		15	76.1 (68.3–84.8)			16	72.9 (62.6–84.8)			31	74.4 (67.6–82.0)		
35	TT	GA	CC		8	80.7 (69.5–93.7)			7	60.4 (48.0–75.9)			15	70.5 (61.3–81.0)		
45	TT	CA	CC		5	77.9 (64.6–94.1)			6	52.9 (41.4–67.8)			11	63.1 (53.7–74.2)		
25	TT	CA	CA		2	71.7 (53.2–96.6)			6	58.9 (46.0–75.4)			8	61.9 (51.1–74.8)		

Bold numbers represent significant P values.

Haplotype combinations were manually inferred and numbered. Homozygote haplotype combinations were numbered 11, 22, 33, 44 and 55. The combinations of the heterozygote haplotypes (12 to 45) were given by one number of each homozygote haplotype e.g. 1 + 2 = 12.

1 *M* major allele, *m* minor allele.

2 Raw geometric mean of serum 25(OH)D concentrations (nmol/L) and corresponding 95%-confidence interval.

3 Adjusted geometric mean of 25(OH)D concentrations (nmol/L) and corresponding 95%-confidence interval. Linear mixed models with family as a random factor, adjusted for age, sex, BMI, holiday, use of solarium, dietary vitamin D intake, use of multivitamin and vitamin D supplements.

adj Adjusted P values. Haplotype combination 44 was excluded in the linear mixed model due to inadequate participants carrying this haplotype combination.

Carriers of haplotype combination 22 encompassing the variant alleles of rs4588 and rs842999 had low serum 25(OH)D concentrations. Conversely, carriers of haplotype combination 11 encompassing the variant allele of rs12512631 had high serum 25(OH)D concentration. Thus, the variant allele of rs12512631 was associated with high low serum 25(OH)D concentrations and the variant alleles of rs4588 and rs842999 were associated with low serum 25(OH)D concentrations. Since the lowest serum 25(OH)D concentrations were observed for haplotype combination 22 carriers, this could indicate that rs4588 is the biologically relevant polymorphism rather than rs842999 since haplotype combination 44 encompassing the C-allele of rs842999 is associated with higher serum 25(OH)D concentrations.

The genetic risk score (range 0–4) was calculated as the sum of the number of A-alleles of rs4588 and C/A-alleles of rs842999 (Figure 1, B). After adjustment for family and confounding factors, we found that an increasing number of risk alleles was associated with lower serum 25(OH)D concentrations. Carriers of no risk alleles had significantly higher serum 25(OH)D concentrations (68.1 (56.2–82.6), 81.0 (64.2–102.2) and 86.5 (70.9–105.5) nmol/L) compared to carriers of all four risk alleles (50.3 (40.3–62.7), 67.5 (53.6–84.9) and 70.1 (57.2–84.8) nmol/L) in both children, adults and all combined, respectively. Overall, there was a mean difference in 25(OH)D concentrations of 35.4, 20.0 and 23.4% between carrying no risk alleles and carrying all four risk alleles in children, adults and all combined, respectively.

**Figure 2:**
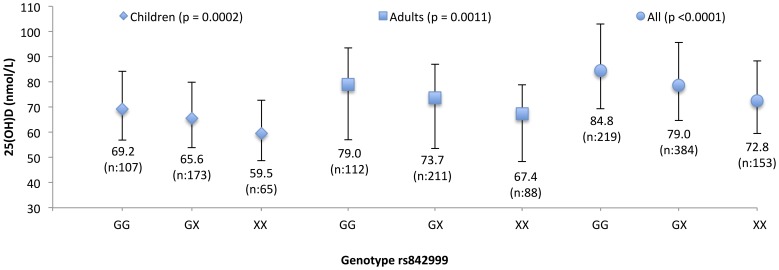
Dose-dependent relationship between genotype GG, GX and XX of rs842999 and serum 25(OH)D concentrations. X-axis stands for genotype GG (GG), GX (GC or GA) and XX (CC, CA or AA) of rs842999. Y-axis stand for serum 25(OH)D (nmol/L). Errors bars stand for 95%-confidence interval and serum 25(OH)D concentrations are given as geometric means. Linear mixed models with family as a random factor, adjusted for age, sex, BMI, ski and sun holidays, solarium use at least once a week, dietary vitamin D intake, multivitamin and vitamin D supplement users was conducted to compare rs842999 genotypes with serum 25(OH)D concentrations. There was a dose-dependent relationship between serum 25(OH)D concentrations and carriers of none, one or two copies of the G-allele. Carriers of two copies of the G-allele, had higher serum 25(OH)D concentrations compared to carriers with only one G-allele or non-carriers in children, adults and all combined, respectively.

For the tri-allelic variant rs842999, there was a dose-dependent relationship between serum 25(OH)D concentrations and carriage of none, one or two copies of the G-allele (Figure 2). Thus, carriers of two copies of the G-allele, had statistically significantly higher serum 25(OH)D concentrations (69.2 (56.8–84.3), 79.0 (62.8–99.4) and 84.8 (69.6–103.4) nmol/L) compared to carriers of only one G–allele (65.6 (53.9–79.9), 73.7 (58.8–92.4) and 79.0 (64.9–96.1) nmol/L) in children, adults and all combined, respectively. The lowest serum 25(OH)D concentrations were observed in non-carriers of the G-allele (59.5 (48.7–72.6), 67.4 (53.8–84.4) and 72.8 (59.7–88.8) nmol/L) in both children, adults and all combined, respectively.

Finally, we made a joint genetic risk score analysis including *CYP2R1* (rs10741657 and rs10766197) and *GC* (rs4588 and rs842999) (Figure 1, C). The genetic risk score (range 0–8) was calculated as the sum of the number of G-alleles of rs10741657, A-alleles of rs10766197, A-alleles of rs4588 and C/A-alleles of rs842999 (Figure 1, C). After adjustment for family and confounding factors, carriers of no risk alleles had statistically significantly higher 25(OH)D concentrations (78.4 (63.6–96.7), 86.3 (66.1–112.7) and 89.0 (72.0–110.0) nmol/L) compared to carriers of all eight risk alleles 43.4 (32.4–58.2), 55.3 (37.5–81.4) and 53.0 (39.6–70.9) nmol/L) in children, adults and all combined, respectively. Overall there was a mean difference in 25(OH)D concentrations of 80.6, 56.1 and 67.9% between carriage of no risk alleles and carriage of all four risk alleles in children, adults and all combined, respectively.

## Discussion

In this present study, we studied the association of 7 prominent vitamin D-related genes with serum 25(OH)D concentrations in 201 Danish families with dependent children in late summer in Denmark, and found that common variants in *CYP2R1* and *GC* genes were statistically significantly associated with serum 25(OH)D concentrations.

The *CYP2R1* gene encodes the key enzyme that converts vitamin D to 25(OH)D in the liver [12] and thus genetic variation in this gene might affect 25(OH)D synthesis. We found that *CYP2R1* variants rs1562902, rs7116978, rs10741657 and rs10766197, were significantly associated with serum 25(OH)D concentrations in both children, adults and all combined. Furthermore, rs10741657-rs7116978, and rs10766197-rs1562902 were in strong LD. The association appeared to be driven by rs10741657 and rs10766197, which are located in the promoter region of the *CYP2R1* gene. We found that non-carriers of rs10741657 and rs10766197 risk alleles had the highest mean serum 25(OH)D concentrations.

Our results are consistent with previous findings. In the study of Wjst et al. [21], rs10766197 was significantly associated with 25(OH)D concentrations in 872 subjects from the German Asthma Family Study. Ramos-Lopez et al. [32] found a statistically significant association between rs10741657 and serum 25(OH)D concentrations in 203 German diabetes families. Two genome-wide association studies (GWAS) of vitamin D concentrations were published in 2010 [24], [25]. Ahn et al. [24] performed a combined meta-analyses in 4,501 subjects from five adult Caucasian cohorts and found that rs2060793, which is in LD with rs10741657 (D´ =  1, r^2^ = 1, HapMap Data Rel 24/phase II Nov 08), was associated with serum 25(OH)D concentrations. Furthermore, these findings were successfully replicated in 2,221 subjects. Wang et al. [25] found that rs10741657 was significantly associated with 25(OH)D concentrations in 30,000 subjects of European descent from 15 cohorts. In the study of Bu et al. [33], rs10741657 and rs10766197 were found to be significantly associated with serum 25(OH)D concentrations in 496 unrelated healthy Caucasian subjects. Lasky-Su et al. [34] conducted a combined analysis in 1,164 subjects from two cohorts of Caucasian and Costa Rica asthmatic children and found that rs10741657 was significantly associated with 25(OH)D concentrations. Zhang et al. [35] found that rs10766197 was significantly associated with 25(OH)D concentrations in 2,897 unrelated healthy Chinese subjects from the Shanghai Osteoporosis Study. In the study of Engelman et al. [36], rs2060793 (in LD with rs10741657 as mentioned previously) was significantly associated with 25(OH)D concentrations in 1,204 women of European descent from the Women’s Health Initiative Observational Study. All the aforementioned studies demonstrate that variants in the *CYP2R1* gene predicts 25(OH)D concentrations.

The *GC* gene encodes the vitamin D binding protein (DBP) that binds and transports blood 25(OH)D and other vitamin D metabolites to their target organs. Less than 0.04% of blood 25(OH)D circulates in free form (bioavailable). Most is bound with high affinity to DBP (83–85%) and with lower affinity to albumin (12–15%) [37]. Variants in the *GC* gene may affect the DBP binding and bioavailability of 25(OH)D and other vitamin D metabolites. Thus, there may be a relationship between phenotype and blood 25(OH)D concentrations.

There is accumulating evidence that variants in the *GC* gene are associated with 25(OH)D concentrations. The most studied *GC*-variants are rs4588 and rs7041, giving three common *GC*-isoforms, *GC1F* (rs7041-T, rs4588-C), *GC1S* (rs7041-G, rs4588-C), and *GC2* (rs7041-T, rs4588-A), which differ by amino acid substitutions and/or by glycosylation (Gozdzik et al. 2011). Several studies have shown that vitamin D status differs significantly depending on rs4588 and/or rs7041 genotype, where the A-allele of rs4588 and the T-allele of rs7041 are consistently associated with lower 25(OH)D concentrations [17], [38]–[45]. In agreement, we found that the A-allele of rs4588 is associated with lower 25(OH)D concentrations. There is biological support that the affinity of both 25(OH)D and 1,25(OH)_2_D is higher for the C-allele of rs4588 than for the A-allele [46]. Based on glycosylation patterns, it is suggested that *GC2* phenotypes that is associated with low vitamin D concentrations should be metabolized faster. Kawakami et al. observed that the metabolic rate was indeed higher in *GC2-2* individuals than in *GC1-1* individuals [47]. In addition, the *GC2* genotype, which is associated with low 25(OH)D concentrations, is also associated with low mean DBP [43]. Strangely, the *GC2* genotype is more frequent in populations living in northern climates [48].

Since the two GWAS studies [24], [25] found a strong association between rs2282679 and 25(OH)D concentrations, there has been increased focus on this polymorphism. Several studies have been published supporting the finding [22], [34], [35], [49]–[51]. The GWAS *GC* variant rs2282679 is in high LD with rs4588. Wang et al. [25] did not include rs4588 because it is not in the HapMap dataset. In one study sample the authors found that rs4588 was in LD with several associated variants from the GWAS study. In the study of Lu et al. [45], rs4588 and rs2282679 (r^2^ = 0.97) were significantly associated with 25(OH)D concentrations in 3,210 Han Chinese. In the study by Berry et al. [52], rs4588 was in strong LD with rs228697 (r^2^ = 0.98), and rs4588 was significantly associated with 25(OH)D concentrations in 6,551 subjects from the British birth cohort. Zhang et al. [35] found that 2282679 and rs4588 were in strong LD in 2,897 unrelated healthy Chinese subjects and the strongest association was observed for rs4588, which accounted for 0.7% of the variation in serum 25(OH)D concentrations. Our results support that rs228697 is in strong LD with rs4588 (Pearson’s r = 0.997, SNAP proxy D’  = 1 r^2^ = 0.98) and that the association with serum 25(OH)D concentrations is most likely driven by rs4588. Zhang et al. [35] argued that it is unlikely that rs2282679 in itself is the disease-causing variant. The possible causal variant is the non-synonymous rs4588, where the C/A base pair change in codon 436 (previously known as 420 [36]) causes a Thr to Lys amino acid substitution. In agreement with Zhang et al. [35] we found that rs4588 was the strongest independent predictor of 25(OH)D concentrations compared to rs2282679. Furthermore, Zang et al [35] found that both the minor T-allele of rs4588 and G- allele of rs2282679 were associated with reduced DBP concentrations. Participants with 3 or 4 risk alleles of the two variants were more likely to have vitamin D concentrations lower than 50 nmol/L (20 ng/mL) compared with non-carriers of the risk alleles.

In our study, several of the significant *GC* variants were in strong LD and the strongest associations with serum 25(OH)D concentrations were observed for rs4588 and rs842999. We observed a dose-dependent relationship between carrying none, one or two copies of the G-allele of the tri-allelic rs842999 and 25(OH)D concentrations. Furthermore, genetic risk score analysis for rs4588 and rs842999 showed that non-carriers of the risk alleles of rs4588 and rs842999 had the highest serum 25(OH)D concentrations.

We made a joint genetic risk score analysis for all four risk variants (*CYP2R1*-rs10741657 and rs10766197, and *GC*-rs4588 and rs842999), and found the largest%-range in mean serum 25(OH)D concentrations (80.6, 56.1 and 67.9%) compared to genetic risk score analysis of *CYP2R1* (rs10741657 and rs10766197; 20.9, 14.1 and 16.5%) or *GC* (rs4588 and rs842999; 35.4, 20.0 and 23.4%) indicating an additive effect. In general, there was a better association between genetic risk score and serum 25(OH)D concentrations in children than in adults. We speculate that the more risk alleles in *CYP2R1* and *GC* genes a subject carries, the more prone the subject will be for having a low serum 25(OH)D concentration. In Denmark, sufficient serum 25(OH)D concentrations are defined as >50 nmol/L [53]. Notably, in late summer in Denmark, where vitamin D status peaks in Danes, children carrying 7 or 8 risk alleles had insufficient serum 25(OH)D concentrations (49.4 and 43.4 nmol/L).

In our study population, none of the investigated SNPs in *CYP24A1, CYP27B1*, *C10orf88* or *DHCR7/NADSYN1* were associated with serum 25(OH)D concentrations. Furthermore, *VDR*-rs731236 was only statistically significant in all combined and rs757343 was statistically significant in children and all combined. False-positive (type 1 errors) results, which are common in studies of the association between genetic markers and outcomes, and the relative small sample size, resulting in statistical reduced power might explain these findings. We consider children and adults as two natural subpopulations due to biological differences, difference in lifestyle, eating patterns and use of multivitamins [28]. We did not use Bonferroni-corrected P-values because a statistically significant association both in children and in adults by itself may be considered a confirmation of an association. A limitation of the study is that the participants’ general vitamin D status relies on a single measurement of serum 25(OH)D concentration. We were not able to calculate the genetic contribution due to the familiar design used in the linear mixed model. A strength of this study is that it is conducted in a healthy Caucasian population and thus the potential impact of diseases is minimized. Furthermore, the blood samples were collected in a relatively small geographical area in Denmark in September to October 2010 and analysed in a single batch with LC-MS/MS with low variation. Furthermore, many known predictors of serum 25(OH)D concentrations were assessed by questionnaire data.

Genetic variants may accelerate or protect against vitamin D deficiency and the genetic effect is life-long. We speculate that individuals with genetically determined low vitamin D concentrations may need different health recommendations in order to improve their serum 25(OH)D concentrations thereby avoiding adverse health outcomes. A study by Engelman et al. [36] found that in women with no risk alleles of rs4588 and rs2060793 (in strong LD with rs10741657 as mentioned previously) who consumed at least 670 IU/d vitamin D all (100%) had 25(OH)D > 50 nmol/L. For women carrying 1, 2 or 3–4 risk alleles and consuming at least 670 IU/d vitamin D, only 84, 72, and 62% had 25(OH)D > 50 nmol/L. Furthermore, the percentage of women with adequate 25(OH)D concentrations rose with each increasing quartile of vitamin D intake. Thus, subjects with genetic predisposition seem to benefit from dietary vitamin D supplementation. In the study by Madsen et al. [28], vitamin D_3_-fortification of bread and milk reduced the decrease in serum 25(OH)D concentrations seen during winter and ensured 25(OH)D >50 nmol/L in healthy Danish families. Whether such a dietary intervention program could ensure adequate serum 25(OH)D concentrations in subjects with genetic predisposition for vitamin D deficiency warrants further study.

## Conclusions

In conclusion, our results support the current evidence that common genetic variation in *GC* and *CYP2R1* may contribute to the variation of serum 25(OH)D concentrations in a healthy population. Notably, genetic risk score analysis revealed that non-carriers of risk alleles of *CYP2R1* rs10741657 and rs10766197, and/or *GC* rs4588 and rs842999 had statistically significantly higher serum 25(OH)D concentrations compared to carriers of all risk alleles.
